# Визуализация надпочечников: в норме и при патологии (обзор литературы)

**DOI:** 10.14341/probl12752

**Published:** 2021-06-07

**Authors:** Т. А. Корб, В. Ю. Чернина, И. А. Блохин, О. О. Алешина, А. В. Воронцов, С. П. Морозов, В. А. Гомболевский

**Affiliations:** Научно-практический клинический центр диагностики и телемедицинских технологий Департамента здравоохранения города Москвы; Научно-практический клинический центр диагностики и телемедицинских технологий Департамента здравоохранения города Москвы; Научно-практический клинический центр диагностики и телемедицинских технологий Департамента здравоохранения города Москвы; Научно-практический клинический центр диагностики и телемедицинских технологий Департамента здравоохранения города Москвы; Национальный медицинский исследовательский центр эндокринологии; Научно-практический клинический центр диагностики и телемедицинских технологий Департамента здравоохранения города Москвы; Научно-практический клинический центр диагностики и телемедицинских технологий Департамента здравоохранения города Москвы

**Keywords:** надпочечники, инциденталомы надпочечников, новообразования надпочечников, диагностическая визуализация

## Abstract

В представленном обзоре рассмотрены нормальная анатомия надпочечников и особенности современных методов их визуализации, которые необходимы для оценки как доброкачественных, так и злокачественных новообразований. В частности, рассмотрены одни из наиболее распространенных образований, такие как аденома, феохромоцитома, метастатическое поражение и адренокортикальный рак. Для этого был проведен анализ релевантных отечественных и зарубежных источников литературы, датируемых сроками с 1991 г. по январь 2021 г.

Во многих случаях образования надпочечников имеют отличительные особенности, которые позволяют охарактеризовать их с помощью неинвазивных методов. В некоторых случаях возможно заподозрить злокачественную природу и вовремя направить пациента на необходимые инвазивные исследования. Компьютерная томография, особенно с применением внутривенного контрастного усиления, представляет собой основной метод визуализации, поскольку в большинстве случаев позволяет предположить нозологическую форму образования. Магнитно-резонансная томография остается высокочувствительным методом с точки зрения выявления опухоли, динамического наблюдения за размерами, однако метод малоспецифичен для определения злокачественного потенциала образования. Позитронно-эмиссионная компьютерная томография также является дополнительным методом и используется в основном в обнаружении злокачественных опухолей, их дифференциальной диагностике, выявлении метастазов и рецидивов после хирургического лечения. Ультразвуковое исследование играет ограниченную роль, тем не менее, имеет большое значение в диагностике у детей, особенно новорожденных. Такие перспективные методы, как радиомика и двухэнергетическая КТ, позволяют расширить возможности визуализации и улучшить диагностическую точность.

Поскольку образования надпочечников часто случайно выявляются при визуализации, выполняемой по другим причинам, важно правильно их интерпретировать. Этот обзор дает читателю широкое представление того, чем различные методы визуализации могут быть полезны при оценке патологии надпочечников и на что следует обращать внимание рентгенологам и врачам-клиницистам.

## Abstract (en)

This literature review focuses on the normal adrenal gland anatomy and typical imaging features necessary to evaluate benign and malignant lesions. In particular, adenoma, pheochromocytoma, metastases and adrenocortical carcinoma were discussed as some of the most common lesions. For this purpose, a review of relevant local and international literature sources up to January 2021 was conducted.

In many cases, adrenal incidentalomas have distinctive features allowing characterization using noninvasive methods. It is possible to suspect a malignant nature and promptly refer the patient for the necessary invasive examinations in some cases. ­Computed tomography, especially with intravenous contrast enhancement, is the primary imaging modality because it enables differential diagnosis. Magnetic resonance tomography remains a sensitive method in lesion detection and follow-up but is not very specific for determining the malignant potential. Positron emission computed tomography also remains an additional method and is used mainly for differential diagnosis of malignant tumors, detecting metastases and recurrences after surgical treatment. Ultrasound has a limited role but is nevertheless of great importance in the pediatric population, especially newborns. Promising techniques such as radiomics and dual-energy CT can expand imaging capabilities and improve diagnostic accuracy.

Because adrenal lesions are often incidentally detected by imaging performed for other reasons, it is vital to interpret such findings correctly. This review should give the reader a broad overview of how different imaging modalities can evaluate adrenal pathology and guide radiologists and clinicians.

## ВВЕДЕНИЕ

Случайно выявленные образования надпочечников более 1 см в диаметре принято называть «инциденталомами» (от англ. «случай», «побочное обстоятельство»). Широкое использование визуализации, включая ультразвуковое исследование (УЗИ), компьютерную томографию (КТ), позитронно-эмиссионную томографию, совмещенную с компьютерной томографией (ПЭТ-КТ), и магнитно-резонансную томографию (МРТ), резко увеличило частоту выявления образований надпочечников. Если у пациента в анамнезе нет злокачественных новообразований и эндокринных нарушений, такие образования в большинстве случаев являются доброкачественными и нефункционирующими аденомами [1]. Тем не менее дифференциальная диагностика доброкачественной и злокачественной природы поражения надпочечников может быть сложной задачей, имеющей решающее значение, особенно для онкологических пациентов.

Частота выявления инциденталом надпочечников, диагностируемых при КТ, составляет до 7% [1–3]. Это может быть связано с высокой распространенностью применения метода, а также возможностью визуализировать надпочечники при исследовании грудной и брюшной полостей. Согласно результатам вскрытий, образования надпочечников являются также одними из наиболее распространенных опухолей, выявляемых не менее чем у 3% людей старше 50 лет [4]. Методы визуализации позволяют не только выявить образования, но и охарактеризовать их морфологическую структуру, при необходимости вовремя направить на инвазивные исследования. Рентгенологи должны помнить о преимуществах и недостатках каждого метода визуализации, избегать неправильной интерпретации, особенно при дифференциальной диагностике доброкачественных и злокачественных образований.

Целью данной работы была оценка особенностей методов визуализации новообразований надпочечников, необходимых для точной характеристики наиболее частых их поражений.

Был проведен анализ релевантных отечественных и зарубежных источников литературы по базам данных eLIBRARY, PubMed, Web of Science с использованием ключевых слов: «надпочечники», «образования надпочечников», «инциденталомы надпочечников», «визуализация надпочечников», «УЗИ», «КТ», «МРТ», «ПЭТ-КТ», «adrenal gland», «adrenal mass», «adrenal incidentaloma», «adrenal imaging», «ultrasonic diagnostics», «CT», «MRI», «PET-FDG». Были проанализированы 80 научных работ за период с 1991 г. по январь 2021 г. В итоговый обзор были включены 57 англоязычных статей, 5 русскоязычных статей. При обнаружении нескольких статей с общей темой и похожими результатами в обзор включалось наиболее позднее исследование.

В данном обзоре рассмотрены нормальная лучевая анатомия надпочечников, особенности методов их визуализации, а также дифференциальная диагностика наиболее частых образований надпочечников (аденома, феохромоцитома, вторичное поражение, адренокортикальный рак).

## НОРМАЛЬНАЯ АНАТОМИЯ НАДПОЧЕЧНИКОВ

Надпочечники располагаются в забрюшинном пространстве в толще околопочечной жировой клетчатки, над верхним полюсом соответствующей почки. Надпочечники состоят из двух морфофункционально самостоятельных эндокринных желез — мозгового и коркового веществ, имеющих различное эмбриональное происхождение.

В период внутриутробного развития плода надпочечники быстро развиваются в течение первых 3 мес. С 12 до 18 нед развития вес надпочечников увеличивается в 7 раз [5]. При рождении размеры их значительно больше, чем у взрослых (примерно от половины до трети размера почек), а вес составляет в среднем 10 г. К 20 годам масса каждого надпочечника увеличивается в 1,5 раза по отношению к массе надпочечника новорожденного и достигает своих максимальных размеров.

По данным УЗИ нормальные надпочечники обычно хорошо видны у новорожденных детей. Это связано не только с большими размерами надпочечников по сравнению с почками, но и с малым количеством забрюшинной жировой ткани и небольшим расстоянием от датчика [6]. При сканировании в В-режиме надпочечники визуализируются с четкими, ровными контурами, треугольной и полулунной формы, с дифференцировкой на однородный гиперэхогенный мозговой и однородный гипоэхогенный корковый слои [7]. У подростков и взрослых обследование надпочечников проводится в нескольких положениях пациента и датчика для оптимальной визуализации. Однако оценка может быть затруднена или невозможна в случае жировой дистрофии печени (нормальная печень является хорошим акустическим окном при оценке правой надпочечниковой области), большого количества газа в кишечнике и желудке (левая надпочечниковая область) и ожирения (плохая проходимость ультразвуковой волны) [6].

На аксиальных срезах КТ и МРТ каждый надпочечник представляет собой структуру вариабельной формы (линейной, треугольной, Y- или V- образной), длиной до 4,5 см, высотой в среднем до 2 см и толщиной около 1 см [8] (рис. 1). С помощью данных методов надпочечники могут быть визуализированы даже у детей первых лет жизни, так как толщина ножек надпочечников превышает толщину ножек диафрагмы.

**Figure:**
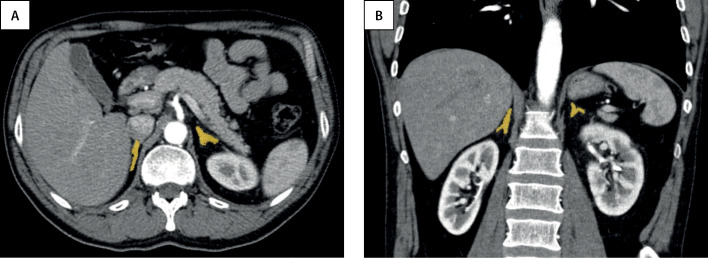
Рисунок 1. Нормальные надпочечники на КТ-изображениях с внутривенным контрастным усилением в аксиальной (A) и корональной (B) проекциях.

## ВИЗУАЛИЗАЦИЯ НАДПОЧЕЧНИКОВ

Ультразвуковое исследование

УЗИ — это метод выбора при оценке состояния надпочечников у новорожденных и детей младшего возраста. Из-за доступности, относительно низкой стоимости и неинвазивности этот метод также часто используется для оценки надпочечников у подростков и взрослых. Кроме того, УЗИ рекомендуется пациентам с артериальной гипертензией или недостаточностью надпочечников, а также при мониторинге опухолей надпочечников, имеющих доброкачественную природу по данным КТ или МРТ. УЗИ с применением B-режима и эластографии позволяет отличить солидную опухоль от кист надпочечников, в отличие от КТ, при которой дифференцировать эти образования возможно только с применением контрастного усиления [6].

Данный метод имеет ряд ограничений. Визуализация надпочечников зависит от таких факторов, как ширина акустического окна, качество оборудования и опыт врача. Сложность визуализации может быть связана с тем, что при оценке данной области отмечается высокий уровень естественного эхосигнала, почти равного уровню окружающей жировой клетчатки [6].

УЗИ с контрастным усилением улучшает визуализацию сосудистого снабжения даже при небольших опухолях надпочечников, но не позволяет дифференцировать злокачественные и доброкачественные новообразования надпочечников [9]. Оценка характера контрастирования в группе доброкачественных поражений надпочечников показала наличие различий между узловой гиперплазией и аденомами. При узловой гиперплазии контрастирование начинается на периферии образования, а при аденомах преобладает смешанный или центральный характер [10].

При обследовании взрослых пороговый средний диаметр поражения надпочечников, доступный для визуализации, составляет 10 мм. Однако в литературе отмечается, что очаговые поражения диаметром 5 мм можно визуализировать через брюшной доступ, а поражения диаметром 2–3 мм — через эндоскопический [11]. Тем не менее некоторые опухоли с максимальным диаметром менее 20 мм в левом надпочечнике могут плохо визуализироваться при трансабдоминальном УЗИ. Это зависит от их локализации в надпочечнике, а также от анатомических и физиологических условий.

Компьютерная томография

КТ является одним из ведущих методов диагностики новообразований надпочечников. КТ является наиболее частым методом при выявлении инциденталом надпочечников, что может быть связано с включением надпочечников в зону сканирования не только при исследовании органов брюшной полости, но и при исследовании грудной клетки. Применение данного метода позволяет не только диагностировать образования надпочечников, но и в большинстве случаев предположить морфологическую природу.

Согласно обновленной версии рекомендаций Американского радиологического общества (ACR, American College of Radiology) от 2017 г. по алгоритму ведения пациентов с инциденталомами надпочечников, о злокачественном потенциале новообразования необходимо судить по размеру поражения, характеру роста при динамическом наблюдении и онкологическому анамнезу (рис. 2) [12]. Так, если инциденталома имеет диагностические признаки доброкачественных новообразований, таких как миелолипома (наличие макроскопического жира), кисты или кровоизлияния (отсутствие накопления контрастного препарата, разница в плотности пре- и постконтрастных изображений до 10 единиц Хаунсфилда (Hounsfield units, HU)), то дообследование или динамическое наблюдение не требуется. При этом было установлено, что даже инциденталомы надпочечников с плотностью больше +10 HU у пациентов без отягощенного онкологического анамнеза в большинстве случаев являются доброкачественными [13].

**Figure:**
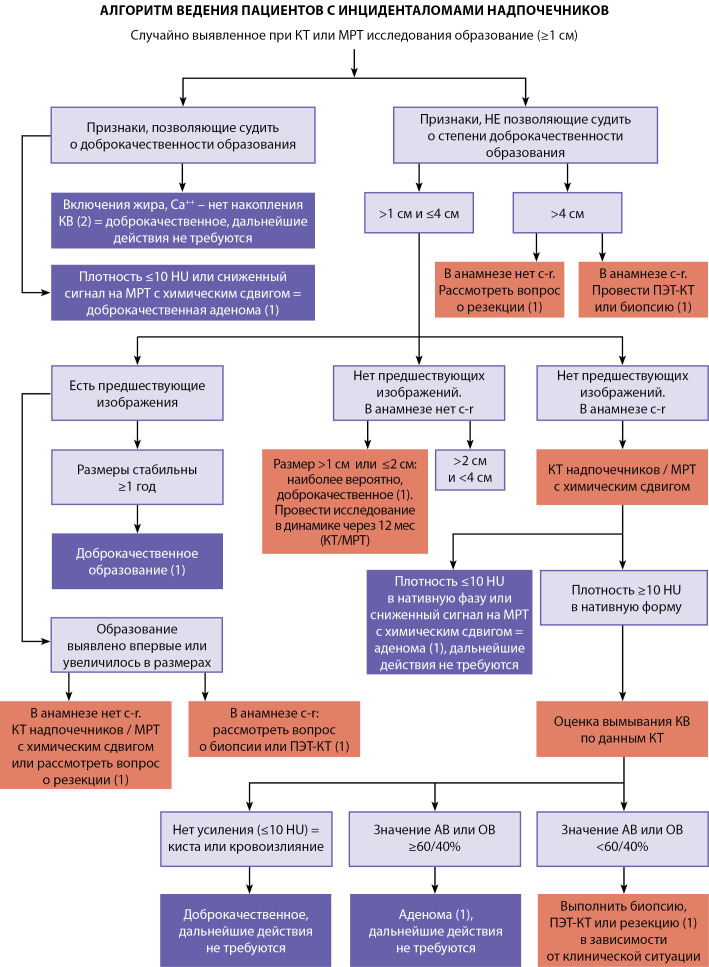
Рисунок 2. Алгоритм ведения пациентов с инциденталомами надпочечников.АВ — абсолютное вымывание; ОВ — относительное вымывание; КВ — контрастное вещество.(Источник: Чернина В.Ю., Блохин И.А., Николаев А.Е. и др. Тактика ведения инциденталом. Раздел 2. Поджелудочная железа, надпочечники, почки / Серия «Лучшие практики лучевой и инструментальной диагностики». — Вып. 36. — М., 2019. — 40 с.)

Образование больше 4 см, не имеющее доброкачественных признаков, необходимо соотнести с онкологическим анамнезом и рассмотреть вопрос о резекции и проведении ПЭТ-КТ с 18F-фтордезоксиглюкозой (18F-ФДГ). В случае если при нативной КТ образование надпочечников по плотности больше +10 HU, целесообразны сканирование с внутривенным болюсным контрастным усилением и использование специального протокола. Рекомендуется протокол с уменьшенной дозой облучения для дальнейшей характеристики образования надпочечников, поскольку он оценивает как плотность, так и характеристики контрастирования в одном исследовании.

Вне зависимости от содержания внутриклеточного жира для доброкачественных аденом характерно быстрое накопление контрастного вещества и быстрое его вымывание. Злокачественные образования могут также быстро накапливать контрастное вещество, но значительно медленнее его вымывают. Поэтому в протоколе важно учитывать получение портально-венозной фазы на 60–90 с и поздней отсроченной фазы на 15-й минуте от момента внутривенного введения контрастного препарата инжектором. В исследовании Sangwaiya M.J. и соавт. было доказано, что отсроченная фаза даже на 10-й минуте показывает низкую чувствительность (76,8%) для характеристики аденом [14]. Соответственно, и применение отсроченной фазы до 5 мин в стандартном протоколе для брюшной полости является недостаточным временем вымывания внутривенного контрастного вещества из доброкачественных образований.

Расчет абсолютного процента вымывания контрастного вещества проводится по формуле:

значение HU в венозную фазу – значение HU в позднюю отсроченную фазу ×100% / значение HU в венозную фазу – значение HU в нативную фазу.

Расчет относительного процента вымывания контрастного вещества проводится по формуле:

значение HU в венозную фазу – значение HU в позднюю отсроченную фазу ×100% / значение HU в венозную фазу.

Расчет показателей абсолютного и относительного процента вымывания контрастного вещества при исследовании надпочечников имеет высокую диагностическую ценность при дифференциальной диагностике аденом от злокачественных образований. Для расчетов в повседневной практике может быть применен специализированный онлайн-калькулятор, разработанный Department of Radiology USC (University of Southern California), доступный по ссылке https://pcheng.org/calc/adrenal_ct.html

Двухэнергетическая КТ (ДЭКТ) является перспективным методом диагностики, который может расширить возможности оценки образований надпочечников и потенциально снизить потребность в дополнительных исследованиях. Метод заключается в переключении напряжения на рентгеновской трубке с последующей дополнительной постобработкой данных. В недавней работе Nagayama Y. и соавт. данный метод позволил точно дифференцировать аденомы надпочечников и метастазы [15].

Следует отметить, что в текущих рекомендациях ACR не учитывался тот факт, что значения HU могут существенно различаться между томографами, а разница может превышать указанные +10 HU [16]. Эти различия зависят от многих факторов, в частности, от производителя и стажа работы аппарата, напряжения рентгеновской трубки, калибровки и толщины срезов при сканировании. Так, результаты исследования Stadler A. и соавт. продемонстрировали, что могут быть существенные расхождения в значениях HU (до 12 HU) при оценке плотности образований надпочечников в зависимости от типа томографа и настроек протокола [17]. Несмотря на то что измерение плотности образований на КТ позволяет в большинстве случаев дифференцировать образования, использование порогового значения HU не может считаться универсальным и влиять на выбор тактики ведения пациентов.

В крупном скрининговом исследовании рака легкого NLST (National Lung Screening Trial) было показано, что из 17 309 пациентов у 419 (2,4%) человек была выявлена патология надпочечников, при этом в 1,2% находка была клинически значимой [18]. В аналогичной программе скрининга рака легких в Италии общая распространенность образований надпочечников была достаточно высокой и составила 4,4% [2]. В условиях большого потока скрининговых исследований рентгенологи могут пропустить инциденталомы надпочечников. Поэтому решением этого вопроса может быть применение алгоритма машинного обучения для помощи рентгенологу в выявлении случайных образований надпочечников.

В течение 2020 г. весь мир был вовлечен в пандемию, вызванную коронавирусной инфекцией (COVID-19). Данное заболевание в первую очередь поражает легкие, в некоторых случаях приводит к дыхательной недостаточности. Однако возможно вовлечение и других органов, включая надпочечники. У пациентов, умерших от тяжелой формы COVID-19, в надпочечниках были выявлены такие изменения, как некроз, жировая дегенерация, кровоизлияние и тромбозы [19]. Это, в свою очередь, влияет на плотность надпочечников и тем самым может затруднять диагностику.

Позитронно-эмиссионная компьютерная томография

ПЭТ-КТ — это комбинированный метод, который помогает дифференцировать доброкачественные образования надпочечников от злокачественных и необходим для выявления рецидива или метастазов у онкологических больных [20]. Результаты метаанализа показывают, что большинство образований надпочечников можно охарактеризовать с помощью ПЭТ-КТ с 18F-ФДГ с высокой чувствительностью (0,97), специфичностью (0,91) и точностью (0,98) [21]. Данный метод позволяет определить точную анатомическую локализацию областей повышенной метаболической активности и произвести их измерения.

Согласно обновленной версии рекомендаций ACR от 2017 г., онкологических больных при наличии образования надпочечника более 4 см или увеличении размеров ранее выявленного образования следует направить на ПЭТ-КТ, так как высока вероятность метастатического поражения [12].

Магнитно-резонансная томография

По данным МРТ нормальные надпочечники в режимах Т1 и Т2 имеют сигнал от низкого до среднего по отношению к сигналу печени и скелетных мышц. В режимах с подавлением сигнала от жира визуализация нормальных надпочечников наилучшая, так как их сигнал гиперинтенсивнее, чем от подавленной жировой ткани. Также это может быть полезно, если образование содержит жировые включения или кровоизлияние. Точность МРТ может быть повышена, если использовать гадолиниевые контрастные препараты. После введения контрастного вещества 90% аденом демонстрируют гомогенное усиление, в то время как 60% злокачественных образований — гетерогенное [22]. Для аденом характерно контрастное усиление в ранний период, однако интенсивность сигнала даже после контрастного усиления в большинстве случаев одинакова как для аденом, так и злокачественных образований, что не является абсолютным диагностическим критерием [22].

Диффузионно-взвешенная визуализация (diffusion-weighted imaging, DWI) — важный дополнительный инструмент при оценке патологических состояний в брюшной полости. Однако с помощью DWI и измеряемого коэффициента диффузии (ИКД) невозможно отличить доброкачественные поражения надпочечников от злокачественных, а также выявить атипичные аденомы [23].

МРТ с химическим сдвигом остается важным инструментом для дополнительной оценки образований надпочечников, выявленных с помощью других методов визуализации, особенно для пациентов с аллергией на йодсодержащий препарат, а также у детей и беременных женщин. Метод заключается в оценке относительной потери сигнала от надпочечника в противофазу по сравнению с фазой, что является достаточным для того, чтобы предположить природу образования. На сегодняшний день используются два способа количественной оценки снижения интенсивности сигнала [22]. Суть первого способа количественной оценки сводится к расчету соотношения сигнала от надпочечника к сигналу другого органа, чаще всего к сигналу селезенки. Данное сплено-адреналовое соотношение (ASR, spleno-adrenal ratio) отражает процент снижения сигнала от образования надпочечника по сравнению с селезенкой и может быть рассчитано по следующей формуле:

(индекс сигнала образования надпочечника в противофазу / индекс сигнала селезенки в противофазу)/

(индекс сигнала образования надпочечника в фазу / индекс сигнала селезенки в фазу) × 100%.

Второй способ количественной оценки снижения интенсивности сигнала заключается в подсчете индекса интенсивности сигнала (SII, signal intensity index), который рассчитывается по формуле:

(индекс сигнала образования надпочечника в фазу – индекс сигнала образования надпочечника в противофазу) / индекс сигнала образования надпочечника в фазу × 100%.

Для расчетов в повседневной практике может быть применен специализированный онлайн-калькулятор, разработанный Department of Radiology USC (University of Southern California), доступный по ссылке https://pcheng.org/calc/adrenal_mri.html

Sebro R. и соавт. провели исследование, доказывающее, что МРТ более чувствительна (в 33% случаев) для обнаружения аденом с низкой плотностью (менее 20–30 HU), чем КТ без контрастного усиления [24]. Но в то же время было показано, что КТ с контрастным усилением превосходит МРТ (100% против 77,5%) [25].

С помощью МРТ возможно отличить доброкачественные новообразования надпочечников, которые содержат внутриклеточный жир (аденома), макроскопический жир (миелолипома) или жидкостной компонент (кисты, псевдокисты). Также метод позволяет провести дифференциальную диагностику между аденомами и метастазами, заподозрить карциному [26].

Радиомика

Радиомика — относительно новая область науки, направленная на получение «невидимых» количественных характеристик медицинской визуализации, основанных на интенсивности, форме, объеме и текстуре. Одним из методов в этой области является текстурный анализ — инструмент для оценки неоднородностей тканей, невидимых человеческому глазу.

Текстурный анализ по данным КТ представляет собой неинвазивный инструмент для характеристики образований надпочечников. Результаты исследования Yu H. и соавт. показали, что некоторые параметры текстуры являются важным критерием в дифференциальной диагностике доброкачественных и злокачественных образований. В частности, такие параметры, как энтропия и стандартное отклонение, имели наиболее высокие показатели чувствительности (до 73–95%) и специфичности (до 86–100%) [27]. Работа Zhang G.M. и соавт. продемонстрировала, что особые параметры текстуры позволяют дифференцировать аденому от феохромоцитомы с точностью до 81% [28]. Эти же авторы в другом своем исследовании сообщили о точности до 77% при отличии метастазов от доброкачественных образований надпочечников [29].

Кроме того, были проведены исследования, где оценивалась точность текстурного анализа по данным МРТ и ПЭТ-КТ для оценки образований надпочечников. Так, комбинированное использование параметра SUVmax и параметров текстурного анализа по данным ПЭТ-КТ может значительно повысить диагностические характеристики для выявления метастазов: чувствительность, специфичность и точность составили 100, 84,6 и 94,3% соответственно [30].

Результаты исследования Schieda N. и соавт. продемонстрировали, что использование текстурного анализа и химического сдвига по данным МРТ также достигает высокой диагностической точности, чем эти методы по отдельности [31]. Чувствительность и специфичность в диагностике метастазов надпочечников при светлоклеточном раке почки от аденом надпочечников составили 93,3% и 86,21% соответственно.

В свою очередь, были опубликованы данные, где сравнивалась эффективность текстурного анализа и данных рентгенологов при оценке образований надпочечников больших размеров (4–10 см) по данным КТ [32]. При этом показатель точности текстурного анализа оказался выше точности показателей рентгенологов (82% против 68,5%).

Возможность получения дополнительных данных при стандартных методах визуализации позволит улучшить диагностическую точность и тактику ведения пациентов с образованиями надпочечников.

## ДИФФЕРЕНЦИАЛЬНАЯ ДИАГНОСТИКА ОБРАЗОВАНИЙ

Аденома надпочечников

Аденома — самая распространенная опухоль надпочечников, частота выявляемости которой составляет 75% среди всех инциденталом [1]. Это доброкачественная опухоль без потенциала злокачественной трансформации, возникающая из коркового слоя надпочечников и состоящая из клеток с внутрицитоплазматическим жиром. Наличие жировых включений является ключевым элементом в дифференциальной диагностике злокачественных новообразований. Большие аденомы могут иметь кистозные компоненты, кальцинаты и геморрагические участки [33].

Типичная аденома имеет размер менее 3 см, что затрудняет ее обнаружение с помощью УЗИ, при котором она выглядит как однородное и гипоэхогенное солидное образование с четкими контурами, гиповаскулярное при цветном допплеровском исследовании и гиповаскулярное при УЗИ с контрастированием [34][35]. Хотя размер не является окончательным показателем доброкачественности, в нескольких исследованиях сообщалось, что средний диаметр аденомы составляет 2–2,5 см, максимально до 3 см. Другие исследования включали более крупные диаметры аденомы (до 4–6 см) [36][37].

При нативной КТ аденома обычно представляет собой четко очерченное округлое или овальное образование, плотность которого равна или немного ниже плотности нормальной ткани надпочечников (до +10 HU, из-за высокого содержания жира) [25].

Для аденомы с низким содержанием липидов требуется проведение КТ с контрастным усилением, где оцениваются такие характеристики, как абсолютное и относительное вымывание контрастного препарата. Если абсолютное вымывание больше 60% или относительное вымывание больше 40% через 15 мин после введения контрастного вещества, то это указывает на аденому. Чувствительность и специфичность этих показателей для дифференциальной диагностики аденомы составляют 88 и 96% для абсолютного вымывания и 83 и 93% для относительного вымывания соответственно [38].

Накопление ФДГ при ПЭТ-КТ является важным критерием при дифференциальной диагностике злокачественных и доброкачественных образований. Однако при аденоме надпочечников может отмечаться повышенное накопление ФДГ, вероятно, обусловленное гормональной активностью этой доброкачественной опухоли [39].

По данным МРТ (независимо от выбранного режима визуализации) аденомы надпочечников имеют четкие, ровные контуры, как правило, однородной структуры, с промежуточной или низкой интенсивностью сигнала по сравнению со скелетными мышцами или печенью; в аденомах больших размеров могут происходить кровоизлияния, что приводит к появлению гиперинтенсивных участков на T1-взвешенном изображении [40].

Важным компонентом МР-протокола надпочечников является химический сдвиг. При использовании этого метода большинство аденом надпочечников демонстрируют потерю интенсивности сигнала на изображениях: снижение интенсивности сигнала более чем на 20% считается диагностическим признаком аденомы [33].

Чувствительность МРТ для аденом плотностью 10–20 HU составляет почти 100%, в то время как для аденом с низким содержанием жира и плотностью больше 30 HU — значительно ниже (13–75%) [41].

Оценка вымывания контрастного вещества при МРТ не демонстрирует такую же диагностическую точность, как при КТ [33]. Таким образом, КТ остается золотым стандартом, особенно при оценке аденом с низким содержанием липидов.

Феохромоцитома

Феохромоцитома происходит из мозгового вещества надпочечников и проявляется избыточной выработкой катехоламинов и связанными с ней клиническими симптомами (головная боль, повышенное потоотделение, учащенное сердцебиение), при этом примерно 10% феохромоцитом протекают бессимптомно [42]. Чаще феохромоцитома является доброкачественной, хотя 10% этих поражений могут быть злокачественными [43]. Злокачественные образования распознаются по локальной инфильтрации или метастазам, обычно поражающим кости, печень, лимфатические узлы, легкие и головной мозг. Размеры феохромоцитом варьируют от 1,2 до 15 см, средний размер — 5,5 см [44].

При подозрении на феохромоцитому первым этапом диагностики является лабораторное обследование. Диагностика феохромоцитомы при визуализации часто является сложной задачей из-за ее разнообразного внешнего вида (связанного с некрозом, фиброзом, кистозной, жировой дегенерацией и кальцификацией), который может имитировать другие заболевания [45].

По данным УЗИ феохромоцитомы неоднородны, инкапсулированы, с гиперваскуляризацией при цветном допплеровском исследовании и ранним артериальным паттерном при УЗИ с контрастным усилением [46].

По данным КТ феохромоцитомы больших размеров могут иметь солидные, кистозные, кальцинированные и/или некротические компоненты. Более мелкие опухоли (менее 7 см) чаще однородной структуры [44]. По данным КТ с контрастным усилением абсолютное и относительное вымывания контрастного вещества аналогичны аденомам. Следовательно, феохромоцитомы невозможно надежно отличить от аденом с помощью данного метода [45][47].

Методом выбора при локализации феохромоцитом является сцинтиграфия c метайодбензилгуанидином (MIBG) [20]. В обзоре литературы Asha Kandathil и соавт. подтверждались высокие чувствительность и специфичность данного метода (94–95%) [48]. Однако авторы представили и противоречивые данные. Так, были продемонстрированы примеры со схожей чувствительностью ПЭТ/КТ и MIBG-сцинтиграфии (76,8% против 75%), а также отдельные случаи большей точности ПЭТ/КТ. В случаях, когда результаты лабораторных исследований не могут поставить точный диагноз, а МРТ противопоказана, метод ПЭТ-КТ может помочь в установке диагноза до гистологической верификации.

По данным МРТ может отмечаться бугристый, полициклический контур опухоли при первично-множественном поражении надпочечника, часто выявляется неоднородность внутренней структуры [40]. В режиме Т2 примерно в 35% случаев феохромоцитомы могут иметь однородный изоинтенсивный или минимально гиперинтенсивный сигнал по отношению к селезенке и в редких случаях (11%) — изоинтенсивный по отношению к спинномозговой жидкости [44]. В режиме Т1 феохромоцитомы обычно изоинтенсивны относительно мышц и гипоинтенсивны относительно печени. На изображениях с противофазой потери интенсивности сигнала не отмечается (в отличие от типичной аденомы) [33]. Однако в некоторых случаях феохромоцитомы могут содержать микроскопический жир, что приводит к потере сигнала на МРТ с химическим сдвигом, имитируя аденомы [43].

Метастазы надпочечников

Многие злокачественные опухоли часто метастазируют в надпочечники, что связано с их богатым кровоснабжением. Наиболее часто в надпочечники метастазирует рак легких, за которым следуют рак молочной железы, толстой кишки, меланома, рак почки и гепатоцеллюлярная карцинома. Метастазы встречаются как односторонние, так и двусторонние, приблизительно в равном соотношении (49 и 51%) [49]. Метастазы клинически не проявляются, но в некоторых случаях (3–8%) обширное двустороннее поражение может вызвать надпочечниковую недостаточность [50]. Метастазы надпочечников не имеют специфических признаков при визуализации по данным КТ без контрастного усиления или МРТ.

По данным КТ метастазы в надпочечниках выглядят как очаговые образования плотностью больше +10 HU, возможны кальциноз и участки кровоизлияния. После введения контрастного вещества наблюдаются неравномерное периферическое накопление и медленное вымывание, что позволяет их дифференцировать от аденом [51].

Недавний метаанализ Wu и соавт. показал важную роль ПЭТ-КТ в дифференциальной диагностике метастазов надпочечников от доброкачественных образований. У пациентов с диагнозом рака легких были продемонстрированы высокие диагностические показатели чувствительности (88,7%) и специфичности (90,8%) [52]. Ретроспективное исследование, проведенное Kim J.Y. и соавт., показало, что такие параметры образований при ПЭТ-КТ, как плотность больше +10 HU и SUV (standardized uptake value, стандартизированный уровень накопления) больше 2,5, более вероятно соответствуют метастазам надпочечников [53]. При этом такие дополнительные данные пациента, как первичный рак в анамнезе, метастазы в другие органы и возраст, значительно влияли на точность диагностики. В исследовании Danae A. Delivanis и соавт. также была доказана высокая специфичность (84%) ПЭТ-КТ и предложено рассматривать этот метод как второй этап визуализации [54].

По данным МРТ особенности визуализации зависят от типа первичной опухоли, но в основном наблюдается низкий сигнал в режиме Т1 и высокий сигнал в режиме Т2; иногда он может быть изоинтенсивным на Т1- и Т2-режимах с кольцевым или неравномерным усилением сигнала после введения контрастного препарата [55]. Химический сдвиг позволяет дифференцировать аденомы надпочечников от метастазов. Однако возможны и ложноположительные результаты при наличии в метастазах внутриклеточного жирового компонента. Так, например, метастазы некоторых первичных опухолей, таких как светлоклеточный почечный рак и гепатоцеллюлярная карцинома, содержат липидные включения [56–58]. Использование DWI также не имеет диагностической ценности для дифференциации аденом от метастазов [59].

Адренокортикальный рак

Согласно рекомендациям Европейского общества эндокринологов (European Society for Endocrinology, ESE) совместно с Европейской организацией по изучению опухолей надпочечников (European Network for the Study of Adrenal Tumours, ENSAT), а также Европейского общества медицинской онкологии (European Society for Medical Oncology, ESMO), пациенты с подозрением на адренокортикальный рак (АКР) должны пройти полное биохимическое обследование надпочечников, а также диагностические методы визуализации [60][61]. Стандартная визуализация для пациентов с подозрением на АКР включает КТ или МРТ брюшной полости, при необходимости КТ грудной клетки или ПЭТ-КТ с 18F-ФДГ. Точное стадирование АКР является решающим шагом в планировании лечения и определении прогноза.

АКР может быть гормонально-активным и проявляться в виде синдрома Кушинга или случайно выявленной гормонально-неактивной опухолью надпочечника. При больших размерах образование может вызывать синдром компрессии и определяться при пальпации.

Внешний вид опухоли зависит от ее размера и на ранних стадиях бывает трудно отличим от аденом. Ввиду быстрого роста АКР характеризуется поздним сроком выявления. Чаще всего выявляется образование больших размеров (в среднем около 10 см), с неровностью контуров, участками некроза и, в некоторых случаях, микрокальцинатами [26][38]. Распространенным осложнением является инвазия в смежные органы, чаще в нижнюю полую вену, в том числе возможно образование внутрисосудистого опухолевого тромба. Следует не забывать о возможности метастатического поражения регионарных лимфатических узлов, а также отдаленных метастазов в печень, легкие и кости.

По данным УЗИ небольшие образования чаще однородны и гипоэхогенны, однако с увеличением размеров увеличивается неоднородность структуры [34]. Нечеткие неровные края образования указывают на агрессивный характер роста. Однако отсутствие этого признака не является критерием доброкачественности [40]. Часто можно выявить паравазальные образования аналогичной опухолевой структуры. При цветном допплеровском исследовании отмечается гиперваскулярность.

При КТ с контрастным усилением выявляется неоднородное накопление контрастного вещества, часто периферическое, в некоторых случаях наблюдается тонкая капсула. Абсолютное и относительное вымывание контрастного препарата при АКР отличается от аденом (менее 60 и 40% соответственно). Однако по данным КТ высокая нативная плотность и неоднородность структуры являются более надежными признаками, чем показатели вымывания, которые варьируют в зависимости от того, какая часть образования был взята для оценки [38].

ПЭТ с 18F-ФДГ необходима для верификации злокачественного потенциала, особенно при опухолях размером более 4 см. Тем не менее, несмотря на способность этого метода дифференцировать доброкачественные и злокачественные образования надпочечников, сохраняется сложность дифференциальной диагностики последних между собой. Радиофармпрепарат 11C-метомидат обладает свойством накапливаться только в тканях коры надпочечников, что позволяет дифференцировать АКР от феохромоцитомы и метастазов [62].

На МРТ-изображениях АКР характеризуется неоднородным сигналом. Участки кровоизлияния могут приводить к появлению гиперинтенсивного сигнала на Т1-взвешенных изображениях, а участки некроза имеют высокую интенсивность сигнала на T2-взвешенных изображениях [26]. На МРТ с химическим сдвигом, как правило, потери сигнала от АКР в противофазу по сравнению с фазой не происходит, однако в редких случаях это не исключено за счет наличия микроскопического жира [41]. МРТ обладает более высокой тканевой контрастностью для определения состояния мягких тканей, чем КТ, что дает ей дополнительное преимущество в оценке границ инвазии опухоли и опухолевого тромба.

Согласно рекомендациям ESE-ENSAT и ESMO, в случае высокой вероятности выявления АКР по данным КТ или МРТ брюшной полости рекомендуется обязательное выполнение КТ органов грудной клетки [60][61]. Дополнительное обследование костей и головного мозга необходимо при клинических признаках отдаленных метастазов. Выполнение пункционной биопсии опухоли надпочечника может быть рекомендовано только при обоснованном подозрении на его метастатическое (вторичное) поражение или в случае неоперабельного метастатического опухолевого процесса перед началом лекарственной противоопухолевой терапии.

## ЗАКЛЮЧЕНИЕ

Визуализация (УЗИ, КТ, ПЭТ-КТ, МРТ) играет ключевую роль в дифференциальной диагностике случайно выявленных образований надпочечников, при целенаправленной предоперационной подготовке пациентов и выборе хирургической тактики. Такие перспективные методы, как радиомика и ДЭКТ, позволяют расширить возможности визуализации и улучшить диагностическую точность. В свою очередь, применение алгоритма машинного обучения поможет снизить у рентгенологов частоту пропусков инциденталом надпочечников, особенно в условиях большого потока скрининговых исследований.
